# Augmentation of antitumour activity of defucosylated chimeric anti-CCR4 monoclonal antibody in SCID mouse model of adult T-cell leukaemia/lymphoma using G-CSF

**DOI:** 10.1111/j.1365-2141.2007.06947.x

**Published:** 2008-01-17

**Authors:** Hiroki Yano, Takashi Ishida, Kazunori Imada, Tomomi Sakai, Toshihiko Ishii, Atsushi Inagaki, Shinsuke Iida, Takashi Uchiyama, Ryuzo Ueda

**Affiliations:** 1 Department of Medical Oncology and Immunology, Nagoya City University Graduate School of Medical SciencesNagoya, Aichi; 2 Department of Haematology and Oncology, Graduate School of Medicine, Kyoto UniversitySakyo-ku, Kyoto, Japan E-mail: itakashi@med.nagoya-cu.ac.jp

Adult T-cell leukaemia/lymphoma (ATLL) has a very poor prognosis (Uchiyama *et al*, 1977; Taylor & Matsuoka, 2005). Because tumour cells from most ATLL patients express CCR4 (Ishida *et al*, 2003), we postulated that this molecule might represent a novel molecular target for immunotherapy and developed a chimeric anti-CCR4 monoclonal antibody (mAb), KM2760 (Niwa *et al*, 2004). Antibody-dependent cellular cytotoxicity (ADCC) is one of the most important mechanisms of action of therapeutic mAb (Ishida & Ueda, 2006). Although ADCC depends on the cytotoxic activity of effector cells, such as natural killer (NK) cells, monocytes/macrophages and neutrophils, these cells are commonly qualitatively suppressed and quantitatively reduced in cancer patients. To overcome this problem, the Fc region of KM2760 is defucosylated, which results in enhanced ADCC (Niwa *et al*, 2004). We have previously reported that a robust KM2760-induced ADCC mediated by autologous effector cells were triggered in some ATLL patients (Ishida *et al*, 2004), whereas little autologous KM2760-induced ADCC was observed in other ATLL patients, especially those with few effector cells in their peripheral blood mononuclear cells (data not shown). These findings prompted us to seek ways in which to augment KM2760-induced ADCC using the immunomodulatory agent granulocyte colony-stimulating factor (G-CSF; Nartograstim, Kyowa Hakko Kogyo Incorporation, Tokyo, Japan), which is commonly used clinically.

**Fig 1:**
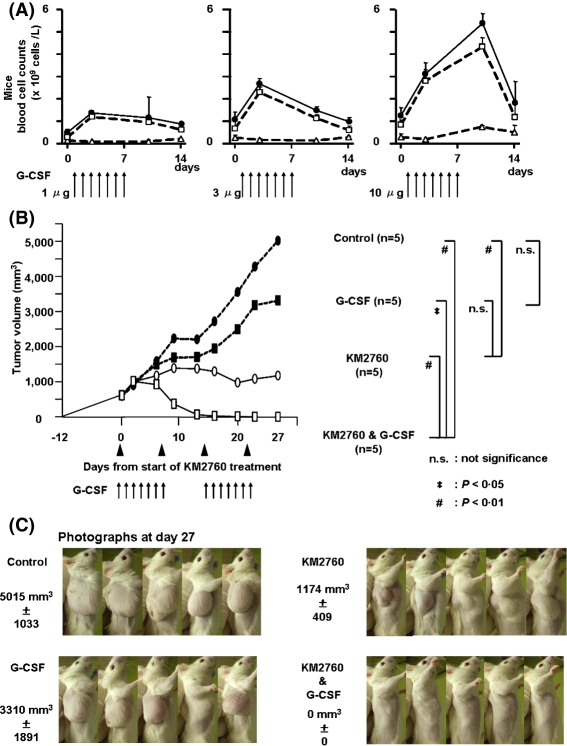
KM2760-induced potent antitumour activity in the Adult T-cell leukaemia/lymphoma (ATLL) tumour-bearing mouse model in the therapeutic setting (A) Total white blood cell, granulocyte and monocyte counts in blood of mice injected with 1, 3 or 10 μg granulocyte colony-stimulating factor (G-CSF) on days 1–7 were measured. Each group consists of three mice, and the blood cell counts are presented as mean + SD (x10^9^ cells/l). Arrows, G-CSF injection; •, total white blood cells; □, granulocytes; and Δ, monocytes. (B) Antitumour activity of KM2760 against pre-established subcutaneous S-YU tumours. Tumour volume was calculated by the following formula: Tumour volume (mm^3^) = 0·5 × (major diameter) × (minor diameter)^2^. Injections of KM2760 or saline were started when tumours were about 609 mm^3^ (12 d after S-YU inoculation). The tumour volume is presented as mean (mm^3^). Arrowheads indicate KM2760 or control injections and arrows indicate G-CSF or control injections. Each group consists of five mice. •, control (saline); ○, KM2760; ▪, G-CSF; and □, KM2760 plus G-CSF. A significant differences between the groups are indicated by #*P* < 0·01 or *P < 0·05. (C) The photographs of each mouse were taken at day 27 from the start of treatment.

Human G-CSF increased granulocyte counts in a dose-dependent manner in severe-combined immunodeficient (SCID) mice (Fig 1A). Subsequently, we evaluated KM2760-induced antitumour activity in the ATLL tumour-bearing mouse model with or without G-CSF in the disseminated setting. The ATLL cell line S-YU (Imada *et al*, 1996) was positive for CCR4 by flow cytometric analysis (data not shown). 1·5 × 10^7^ S-YU cells were inoculated s.c. into SCID mice. The tumour-bearing mice were divided into four groups of five each for treatment with KM2760 or control (saline), KM2760 plus G-CSF or G-CSF alone, to ensure that the mean tumour volumes were same in each group. KM2760 (20 mg/kg) or saline injections into the tail veins of the mice were started 12 d after tumour inoculation, and continued weekly for 4 weeks. The significance of differences in the tumour volume among the groups was estimated by the Mann–Whitney *U*-test. In this study, *P* < 0·05 was considered significant. G-CSF (10 μg/mouse) was injected s.c. into SCID mice on days 0–6 and 14–20 from the start of KM2760 treatment. Mean tumour volumes in KM2760 recipients and control mice 27 d after the start of KM2760 treatment were 1174 ± 409 and 5015 ± 1033 mm^3^ respectively (*P* = 0·0090). KM2760 combined with G-CSF treatment yielded an even more robust antitumour effect, with all recipients achieving complete remission, i.e. subcutaneous S-YU tumours completely vanished. The difference between the tumour volume in animals treated with KM2760 plus G-CSF and those with KM2760 alone was highly significant (*P* = 0·0053) (Fig 1B and C). None of the mice in the present study showed evidence of toxicity that could be attributed to the KM2760 and/or the G-CSF injections. These findings indicate that human G-CSF significantly augments the antitumour activity of KM2760 in the ATLL tumour-bearing mouse model.

**Fig 2:**
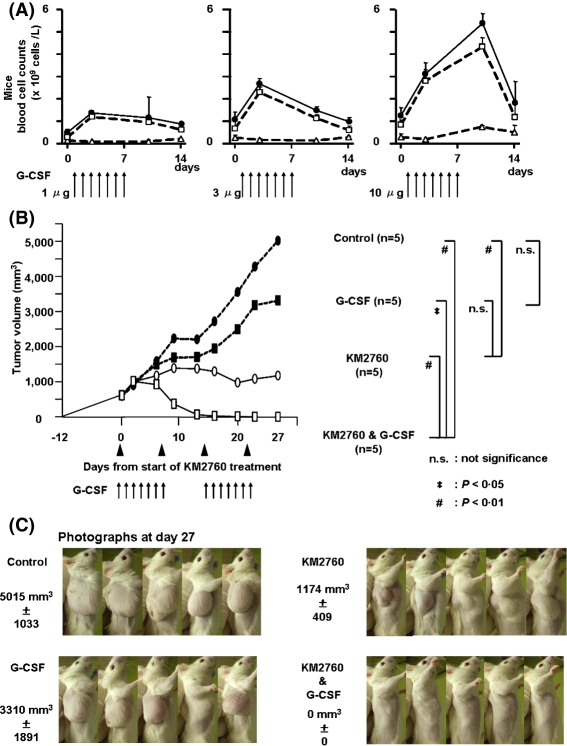
Granulocyte colony-stimulating factor (G-CSF)-enhanced KM2760-induced phagocytosis mediated by mouse monocytes/macrophages (A) Mouse monocytes/macrophages phagocytosis of S-YU cells in the presence or absence of KM2760. Rituximab was the control chimeric mAb. Pooled monocytes/macrophages from peritoneal exudate cells of 14 naïve or 15 G-CSF-injected severe combined immunodeficient (SCID) mice were used for phagocytosis assays. The E/T ratio was fixed at 5, and the incubation period was 1 h. Flow cytometric analyses of PKH26-labelled S-YU cells stained with phycoerythrin cyanin 5 (PECy5)-conjugated anti-F4/80 mAb are shown. (B) The percentage of phagocytosis for each group is indicated as a bar graph. (C) The formula to calculate the percentage phagocytosis of S-YU is illustrated. S-YU cells phagocytosed by monocytes/macrophages are indicated in the upper right quadrant as R2.

Next, we explored the mechanisms whereby human G-CSF augments the anti-ATLL activity of KM2760 *in vivo*. Because KM2760 mediates only ADCC and not complement-dependent cytotoxicity or direct anti-proliferative activity (Ishida *et al*, 2004), it was hypothesized that G-CSF augmented KM2760-induced ADCC *in vivo*. Standard 4-h ^51^Cr-release assays were performed using pooled granulocytes from 14 G-CSF-injected or 15 naïve SCID mice as effector cells (E) and S-YU cells as targets (T) at E/T ratios of 50:1, 100:1 and 200:1. However, KM2760 (10 μg/ml) failed to induce any ADCC against S-YU cells in the presence of granulocytes from either G-CSF-injected or naïve SCID mice at any E/T ratio (data not shown). We next investigated the phagocytic activity of G-CSF-primed monocytes/macrophages, because we recently reported the importance of the innate monocyte/macrophage network, not the NK cell network, to deplete target cells through FcR-dependent pathways during antibody-based immunotherapy in mice (Uchida *et al*, 2004; Ishida *et al*, 2006). Monocytes/macrophages were prepared from peritoneal exudate cells of 14 SCID mice receiving 10 μg G-CSF twice (at 20 h and 5 h before testing) or 15 naïve SCID mice. As S-YU does not express CD20, rituximab (Chugai Pharmaceutical Co., Ltd, Tokyo, Japan) was used as a control mAb for KM2760. PKH26 (Sigma-Aldrich, St Louis, MO, USA) labelled S-YU (1 × 10^4^) and pooled monocytes/macrophages from 14 G-CSF-injected or 15 naïve SCID mice (5 × 10^4^) were co-cultured with or without KM2760 or rituximab at a final concentration of 10 μg/ml for 1 h. The cells were stained with phycoerythrin cyanin 5 (PECy5)-conjugated anti-F4/80 mAb (Serotec Ltd, Oxford, UK), which specifically binds mouse monocytes/macrophages, and analysed by BD FACSCalibur Flow Cytometer (BD Biosciences, San Jose, CA, USA) as previously described (Ishida *et al*, 2006). The percentage of spontaneous, rituximab-induced, and KM2760-induced phagocytosis of S-YU mediated by pooled monocytes/macrophages from 15 naïve SCID mice was 8·6%, 13·4% and 21·5% respectively, whereas these values from 14 G-CSF primed SCID mice were 19·2%, 17·8% and 30·6% respectively (Fig 2A and B). The formula used to calculate the percentage of phagocytosis of S-YU is shown in Fig 2C. These findings indicate that human G-CSF augments not only natural phagocytosis but also KM2760-induced phagocytosis of S-YU by mouse monocytes/macrophages, although the underlying mechanisms require further clarification. The data presented here are the first to address the potential G-CSF-induced augmentation of monocytes/macrophages in the antitumour activity of a therapeutic mAb in an *in vivo* animal model.

In conclusion, the present study is the first to report that antitumour activity induced by next-generation defucosylated therapeutic mAb (Carter, 2006; Ishida *et al*, 2006) was strongly augmented by the immunomodulatory agent G-CSF *in vivo*. This finding has encouraged us to conduct of a phase-I clinical trial of the completely defucosylated anti-CCR4 mAb in patients with CCR4-positive T-cell lymphomas including ATLL (ClinicalTrials.gov Identifier: NCT00355472) to develop a novel treatment strategy for patients with ATLL. In the near future, the efficacy of combination treatments including anti-CCR4 mAb and immunomodulatory agents against refractory ATLL will be assessed in clinical trials.
